# Severe Malaria in Angola: The Clinical Profile and Disease Outcome Among Adults from a Low-Endemic Area

**DOI:** 10.3390/biomedicines12112639

**Published:** 2024-11-19

**Authors:** Inês Morais, Soraia Rodrigues, Aida Mas, Serguei Escalon, Adalzira Borrego, Fatima Nogueira, Maria Lina Antunes

**Affiliations:** 1 Global Health and Tropical Medicine (GHTM), Associate Laboratory in Translation and Innovation Towards Global Health (LA-REAL), Instituto de Higiene e Medicina Tropical (IHMT), Universidade NOVA de Lisboa (UNL), Rua da Junqueira 100, 1349-008 Lisboa, Portugal; 2 Instituto de Higiene e Medicina Tropical, Universidade NOVA de Lisboa, Rua da Junqueira 100, 1349-008 Lisboa, Portugal; 3 Faculdade de Medicina da Universidade Agostinho Neto (FMUAN), Rua Principal da Camama, Distrito Urbano da Cidade Universitária, Talatona CP 815, Luanda, Angola; 4 Hospital Central de Lubango Dr. António Agostinho Neto (HCL), Rua Dr. António Agostinho Neto, Bairro Arco Íris, Lubango CEP 244, Huíla, Angola

**Keywords:** severe malaria, Angola, *Plasmodium falciparum*, renal impairment, impaired consciousness

## Abstract

Background/Objectives: Severe malaria poses a significant public health concern in Angola, particularly among adults. This study assessed the clinical manifestations and outcomes of severe *Plasmodium falciparum* malaria in adult patients admitted to Hospital Central Dr. António Agostinho Neto of Lubango (HCL), Angola. Methods: The study retrospectively reviewed medical records of patients over 14 years old admitted with severe malaria during the first quarter of 2021 and 2022, coinciding with the peak transmission season. The World Health Organization (WHO) criteria were used to clarify the disease severity. The cohort included 640 patients—167 in 2021 and 473 in 2022—distributed across the following departments: the Intensive Care Unit (ICU; *n* = 81), Medicine (MED; *n* = 458) and Infectiology (INF; *n* = 101). Results: The median age was 26 years and 59.4% were males. Renal impairment was the most frequent severe manifestation, affecting 37.4% of cases. The mortality rate across the study period was 7%, showing a notable decrease from 10.2% in 2021 to 5.9% in 2022. The higher mortality rate in 2021 may reflect the impact of the COVID-19 pandemic, which limited hospital access and delayed care, resulting in more critical cases being admitted at a later stage. In 2022, with reduced COVID-19 pressures, earlier access to treatment may have improved outcomes, contributing to the lower mortality rate. Conclusions: This study emphasizes the need to assess the clinical burden of severe malaria in low-endemic regions, where shifting patterns may signal emerging threats such as antimalarial drug resistance. Further research is essential to optimize control strategies and strengthen surveillance systems, reducing morbidity and mortality.

## 1. Introduction

In 2022, nearly half of the world’s population remained at risk of malaria, with an estimated 249 million cases and 608,000 deaths [1]. The WHO African Region accounted for 95% of global malaria cases and 96% of malaria-related deaths [1]. Among African nations, Angola ranked fifth in malaria cases and seventh in malaria-related deaths [1]. Malaria incidence varies significantly across Angolan provinces, categorized into three epidemiological zones: perennial high transmission in the north, mesoendemic in the central region, and seasonal (unstable) in the south, including Huíla Province, where Lubango, the capital, is located [2,3].

Malaria symptoms are non-specific, and clinical diagnosis relies on the presence of fever or a history of fever. Severe malaria is associated with a significantly increased risk of death compared to uncomplicated malaria, with more than 90% of severe malaria cases attributed to *Plasmodium falciparum* [4,5]. It is defined by both clinical and laboratory criteria, including impaired consciousness, prostration, acidosis, hypoglycemia, severe anemia, renal impairment, jaundice, pulmonary edema, significant bleeding, shock, and hyperparasitemia [4,5].

In regions with low endemicity, such as Huíla Province [2,3], severe malaria frequently affects young adults due to lower immunity from reduced exposure, increasing susceptibility to severe forms of the disease [6,7]. Furthermore, malaria outbreaks in these low-transmission areas can exacerbate the severity of cases [8,9]. Despite the significant disease burden, there is limited published research focusing on severe malaria in African adults, particularly in low-endemic areas [8,9].

During 2021 and 2022, the COVID-19 pandemic placed significant strain on Angola’s healthcare system, further complicating the response to malaria. The country faced challenges in managing both public health crises, as resources were often redirected to manage the spread of the coronavirus, which led to disruptions in malaria prevention and treatment services [10,11].

This study aimed to describe the clinical presentation and outcomes of severe malaria patients admitted to Hospital Central Dr. António Agostinho Neto of Lubango (HCL), Huíla Province, Angola. Patients were treated in the hospital’s Intensive Care Unit (ICU), Infectiology (INF), and Internal Medicine (MED) departments. Additionally, the study sought to compare the characteristics of survivors and non-survivors and evaluate potential differences in clinical features and outcomes between the two study periods, 2021 and 2022, to identify trends and factors influencing mortality.

## 2. Materials and Methods

### 2.1. Study Site

**Figure 1:**
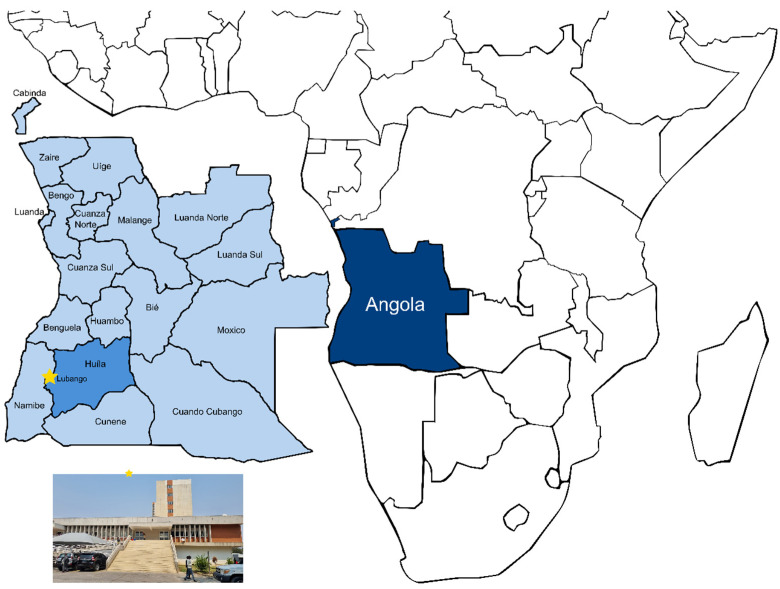
Map of Angola provinces highlighting the study site. Dark blue, Angola; light blue, Angola’s provinces; medium blue, Huíla Province; star, Lubango city; HCL, Hospital Central do Lubango Hospital Central Dr. António Agostinho Neto. The figure was created and edited using Inkscape software (version 1.2, Inkscape Project, Boston, MA, USA).

A retrospective descriptive hospital-based study was conducted at HCL, located in Lubango, the capital of Huíla Province, Angola (Figure 1). HCL is a level 3 healthcare unit, one of the largest in southern Angola, and serves not only Huíla Province, but also the neighboring provinces of Namibe and Cunene. The hospital handles over 70,000 medical consultations annually, including external consultations. The study aimed to capture data during the peak malaria transmission period in Huíla Province, which was reported to occur between January and March [3].

### 2.2. Patients

This study analyzed individual patient records (including demographic information, presenting signs and symptoms, laboratory examinations, and outcomes) from those admitted to HCL from January to March in 2021 and 2022. These months represent the peak malaria transmission season in the region [3]. Patients were identified through hospital admission records to the Departments of Internal Medicine (MED), Infectiology (INF), and the Intensive Care Unit (ICU), with no severe malaria cases reported in other departments. Inclusion criteria for the study were as follows: patients of all genders, being at least 14 years of age, and a laboratory-confirmed diagnosis of *P. falciparum* infection through a rapid diagnostic test (RDT) or a positive blood smear. HCL only admits patients 14 years of age or older. In the study period, all patients admitted to HCL with malaria exhibited one or more of the criteria defined by the WHO for severe malaria. For epidemiological purposes, severe malaria was defined according to the World Health Organization [4] by the following criteria: impaired consciousness, prostration, multiple convulsions, hypoglycemia, anemia, severe renal impairment, jaundice, pulmonary edema, significant bleeding, shock, hyperparasitemia, and metabolic acidosis (criteria detailed in Appendix A) [4].

### 2.3. Statistical Analysis

Data collection and the construction of the study database were conducted by a medical doctor and a nurse (both with professional clearance to consult individual patient records), registered in a Microsoft Excel Database (version 16.0, Microsoft Corporation, Redmond, WA, USA) and analyzed using GraphPad Prism software (version 8.0, GraphPad Software, San Diego, CA, USA). The chi-squared test was primarily used to compare the prevalence of severe malaria criteria between survivors and non-survivors, while Fisher’s exact test was employed when sample sizes were small or when the chi-squared test assumptions were not met to ensure accurate *p*-value estimation. Mann–Whitney tests were used to compare median SOFA scores from the 2021 and 2022 groups and to assess age differences between survivors and non-survivors.

### 2.4. Research Ethics

The study was approved by the Hospital Central Dr. António Agostinho Neto (HCL) institutional ethics review committee (Proc. n° 02/2023).

## 3. Results

### 3.1. Characteristics of Patients

This retrospective study included adult patients with severe malaria hospitalized at HCL between January and March in 2021 and 2022.

Of the 640 patients included in this study, 93.3% (597/640) were confirmed to be infected with *P. falciparum* through thick blood film and 6.7% (43/640) tested positive with a rapid diagnostic test (RDT) alone. In 2021, 167 patients were included, and in 2022, the number increased to 473. From the cohort of 640 patients, a total of 81 patients (12.7%) were admitted to the ICU, 101 (15.8%) to INF, and 458 (71.5%) to MED. January was the month with the highest number of hospitalizations in both years (Figure S1 of the Supplementary Materials).

Females represented 40.6% (260/640) of the cohort, while males accounted for 59.4% (380/640) of patients included in this study. The male/female ratio in the ICU was 4:1 across both years, while in INF it was 7:3 in 2022 (Table 1). Recognizing the potential impact of age on malaria-related mortality [10], the patients were divided into three different groups: 14–18 (adolescents), 18–60 (adults), and ≥60 years (seniors) [8,10].

The median age of the study population was 26 years (mean of 30.3 ± 14.7), with a range from 14 to 88 years. Adults aged 18–59 (adults) made up 81% (517/640) of the cohort, patients under 18 (adolescents) accounted for 13% (83/640), and seniors aged 60 and above comprised 6% (40/640) of the study participants (Table 1). The average time from symptom onset to hospital admission was 4.4 days (±2.1 days), based on patient reports.

### 3.2. Clinical Manifestations of Severe Malaria Patients

**Figure 2:**
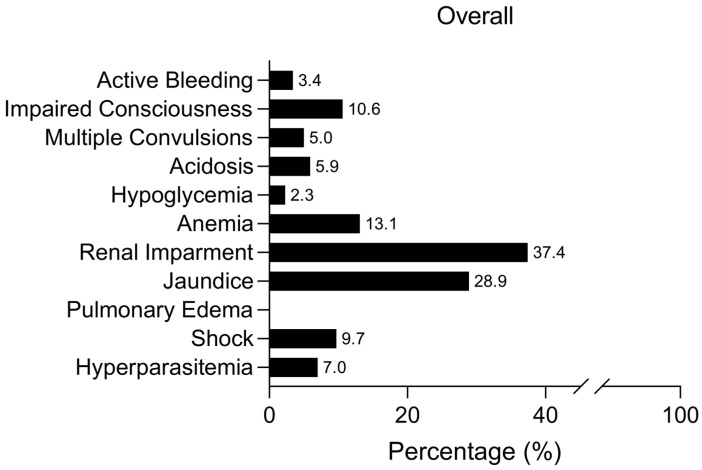
Overall severe malaria profile of patients included in this study. Numbers beside bars represent the % of patients that presented the correspondent severe malaria criteria. Pulmonary edema was not recorded in any of the patients.

**Figure 3:**
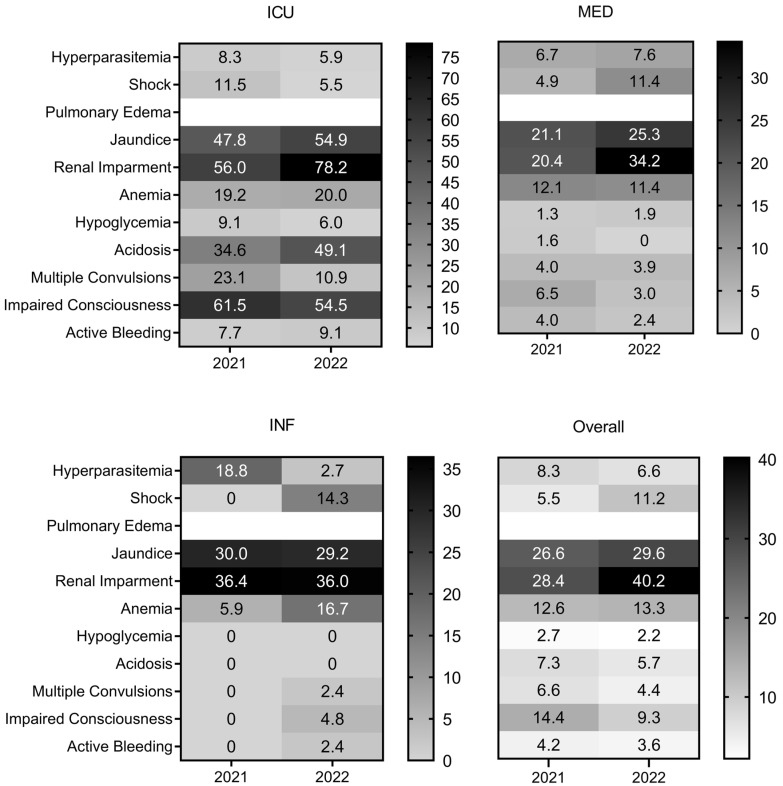
Severe malaria profile of patients admitted to HCL and included in this study during 2021 and 2022. Numbers inside boxes represent the % of patients that presented the correspondent severe malaria criteria admitted to each department: ICU, Intensive Care Unit; INF, Infectiology; MED, Internal Medicine; Overall, aggregated data from all three departments. Pulmonary edema was not recorded in any of the patients.

All 640 patients in this study tested positive for *P. falciparum* and met one or more of the WHO criteria for severe malaria [4]. Pulmonary edema was not observed in any of the enrolled patients. Across both years and all departments, renal impairment emerged as the most prevalent severe malaria manifestation, affecting 37.4% (209/559) of all patients (Figure 2). Jaundice was the second most common symptom, observed in 28.9% (157/544) of cases overall (Figure 2). The data highlighted a notable increase in the percentage of patients presenting severe malaria symptoms in 2022 compared to 2021, potentially due to improved case reporting and higher admission numbers. Critical indicators such as impaired consciousness and acidosis were most frequently reported among ICU patients, whereas fewer severe signs were observed in patients admitted to MED and INF (Figure 3). Despite this, severe outcomes, including active bleeding and impaired consciousness, were still reported in these departments, underscoring the varied severity profile across hospital departments (Figure 3).

The absence of multiple convulsions, active bleeding, impaired conscientiousness, or shock in patients admitted to INF in 2021 was likely due to the lower admission rates that year compared to 2022. As expected, severe malaria manifestations were less frequent in patients admitted to MED and INF compared to those in the ICU. However, some critical signs, such as impaired consciousness and active bleeding, were observed among patients in MED and INF (Figure 3). In the ICU, more than 56% (14/25) of patients suffered from renal impairment in 2021, which increased to 78.2% (43/55) in 2022 despite the overall rise in admissions. Renal impairment rates remained consistently high (above 20%) among those admitted to MED and INF across both years (Figure 3). A detailed description of the clinical features of all patients during the study period is provided in Table S1 (Supplementary Materials).

The most prevalent clinical symptoms among ICU patients were renal impairment, jaundice, acidosis, and impaired consciousness, with a median Glasgow coma score (GCS) of 10 (min. 4), and 25% of the patients had a GCS ≤ 8. Disease severity was assessed using the Sequential Organ Failure Assessment (SOFA) score in ICU patients, which showed a median score of 11 (range 4–21). Although the median SOFA score increased from 2021 (10.5, range 4–18) to 2022 (11.0, range 6–21), this difference did not reach statistical significance (*p* = 0.83).

### 3.3. Clinical Manifestations and Outcome

During this study, 45 patients died from complications of severe malaria, 10.2% (17/167) in 2021 and 5.9% (28/473) in 2022. A chi-squared test with Yates’ continuity correction (X-squared = 2.81, df = 1, *p* = 0.09) indicated that this difference in mortality rates between the two years was not statistically significant. Among these patients, 44.4% (20/45) were female and 55.6% (25/45) male, with an average age of 28.9 ± 14.1 years (ranging from 14 to 85 years; survivors’ age ranged from 14 to 88 years). A chi-squared test (X-squared = 0.15, df = 1, *p* = 0.70) indicated no statistically significant difference in mortality between males and females, suggesting that sex was not a significant factor in the mortality outcomes for this cohort. No significant difference in age was found between patients who died from complications of severe malaria, with an average of 28.9 ± 14.1 years and those who survive to an average age of 30.3 ± 14.7 years (*p* = 0.33; Mann–Whitney test) and a relationship was found between age and mortality in survivors. A detailed description of the clinical features of the deceased patients is provided in Table S2 (Supplementary Materials). The median length of stay (LOS) was 7 days in 2021 (range of 1–52) and 8 days in 2022 (range of 1–55). The median SOFA score was higher in the non-survivor group (median = 12, range of 7–21) compared to the survivor group (median = 10.5, range of 4–15). However, a Mann–Whitney test indicated that this difference in SOFA scores between survivors and non-survivors was not statistically significant (*p* = 0.32). This suggests that, although the median SOFA scores were higher in non-survivors, the observed difference did not achieve statistical significance.

A detailed comparison of severe malaria criteria between survivors and non-survivor patients across all departments (the ICU, MED, and INF) is presented in Table 2. Renal impairment was significantly more prevalent in non-survivors (76.7%, 33/43) compared to survivors (37.4%, 176/470), with a Fisher’s exact test *p*-value of <0.001 and an odds ratio of 6.35 (95% CI: 2.97–14.80). Metabolic acidosis showed an even greater disparity, affecting 52.6% of non-survivors (20/38) versus 7.5% of survivors (18/595) (*p* < 2.2 × 10^−16^; OR = 30.02, 95% CI: 13.07–70.58). Impaired consciousness was observed in 42.2% of non-survivors (19/45) compared to 10.6% of survivors (49/595) (*p* < 0.001; OR = 8.09, 95% CI: 3.94–16.46). Jaundice affected 40.0% of non-survivors (14/35) and 28.9% of survivors (143/495), but this difference was not statistically significant (*p* = 0.175; OR = 1.70, 95% CI: 0.78–3.63). Hyperparasitemia did not show a significant association with mortality. It was present in only 4.8% (2/42) of non-survivors and 7.2% (38/529) of survivors. Statistical analysis using both the chi-squared test and Fisher’s exact test confirmed that the difference was not significant (*p* = 0.78 and *p* = 0.76, respectively). The odds ratio was 0.65 (95% CI: 0.07–2.67), indicating that hyperparasitemia was not a predictor of mortality in this cohort. Shock also showed minimal variation between non-survivors and survivors, with no significant association found (*p* = 0.61; OR = 0.65, 95% CI: 0.12–2.12). Multiple convulsions were significantly associated with higher mortality in this cohort. They were observed in 13.3% (6/45) of non-survivors compared to 4.4% (26/595) of survivors. Fisher’s exact test indicated a significant association (*p* = 0.019) and an odds ratio of 3.36 (95% CI: 1.07–9.01), suggesting that patients experiencing multiple convulsions were more than three times as likely to die compared to those without them. Hypoglycemia and bleeding also emerged as strong predictors of mortality. Hypoglycemia was present in 12.2% (8/45) of non-survivors versus 1.4% (6/430) of survivors. This difference was highly significant (*p* < 0.001, Fisher’s exact test), with an odds ratio of 15.08 (95% CI: 4.33–55.80), indicating that patients with hypoglycemia were 15 times more likely to succumb to the disease compared to those without this condition. Bleeding was detected in 24.4% (11/45) of non-survivors compared to only 1.8% (11/595) of survivors, demonstrating a highly significant association with mortality (*p* < 0.001, Fisher’s exact test) and an odds ratio of 16.97 (95% CI: 6.20–46.72). Thrombocytopenia was present in 82.2% of survivors and 75.6% of deceased patients and there was a tendency for platelet counts to decrease as parasitemia increased (Supplementary Figure S2).

**Figure 4:**
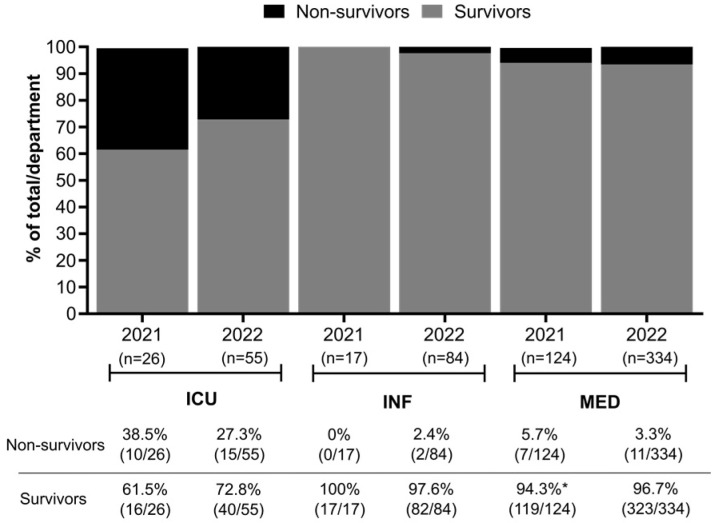
Clinical outcome of severe malaria patients. * Three (3) patients admitted to MED during 2021 abandoned the hospital before receiving medical discharge.

As expected, the ICU had the highest mortality rates in both years, 38.5% (10/26) in 2021 and 27.3% (15/55) in 2022 (Figure 4), and 68% of the patients died within 48 h of admission. None of the patients admitted to INF in 2021 were non-survivors (the 2021 numbers are probably biased due to fewer patients having been admitted to INF during 2021 as compared to 2022; Figure 4). The ICU also accounted for the majority of fatalities in both years. The overall mortality rate was reduced by half from 2021 (10.2%; 17/167) to 2022 (5.9%; 28/473) (Figure 4), although the total number of patients was higher in 2022.

**Figure 5:**
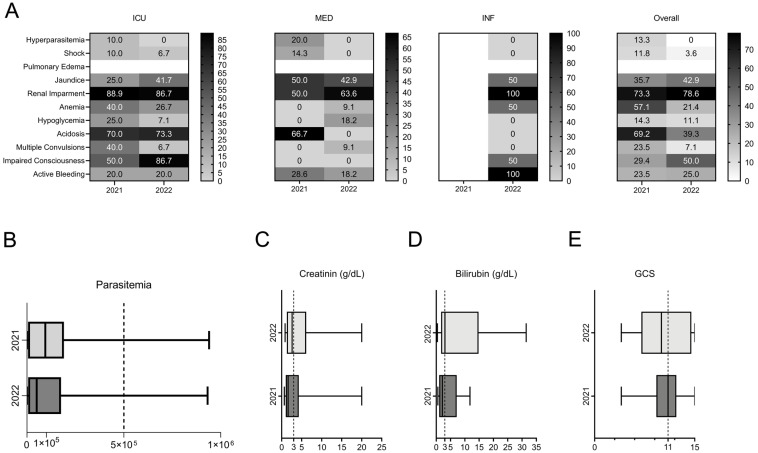
Severe malaria features on admittance amongst non-survivors. (**A**) Numbers represent the % of patients admitted to each department: ICU, Intensive Care Unit; INF, Infectiology; MED, Internal Medicine; Overall, aggregated data from all three departments. Pulmonary edema was not recorded in any of the patients. There were no fatalities in INF during 2021. (**B**) Parasitemia at admission distributed by department and outcome. Black bars, non-survivors; white bars, survivors. (**C**,**D**) Creatinine and bilirubin levels and (**E**) Glasgow coma score (GCS). Dotted line indicates cut-off values for each parameter.

Of those who died, 48.8% (20/415) had acidosis and 76.7% (33/43) presented renal impairment at admission (Supplementary Table S2). Impaired consciousness was the third most common sign, affecting 42% (19/45) of non-survivors. Renal impairment, impaired consciousness, and jaundice emerged as the most commonly observed clinical features amongst non-survivors, with an increasing occurrence from one year to the next (Figure 5A). Overall, the median parasite density was 120,622 parasites/µL (Figure 5B).

In 2022, non-survivors presented higher levels of creatinine and bilirubin (median of 2.62 and range of 0.86–20.00 and median of 3.04 and range of 0.40–31.46, respectively) compared to 2021 (median of 1.63 and range of 0.66–20.00 and median of 2.14 and range of 0.56–11.79, respectively) (Figure 5C,D). Impaired consciousness (cerebral malaria, unarousable coma with GCS < 11) [11] was registered in 29.4% (5/17) of patients in 2021 (median GCS = 11, min. 4) and in 50% (14/28) in 2022 (median GCS = 10, min. 4; Figure 5E). All patients admitted to MED who were non-survivors presented a GSC > 11 (ranging from 12 to 15) at admission. In INF, the two patients who died in 2022 had a GCS of 6 and 15 on admission. There was a significant association between cerebral malaria (impaired consciousness with GSC < 11) at admission and mortality (*p* < 0.0001; chi-squared test); 42.2% (19/45) of those who died presented a GCS < 11 upon admission.

## 4. Discussion

This study provides an overview of the clinical characteristics of severe malaria patients admitted to HCL (Lubango, Huíla Province, Angola) during the peak months of January, February, and March in 2021 and 2022. This research represents the first comprehensive systematic characterization of severe malaria in adult patients in Angola, expanding upon prior research that included only 101 patients (Antunes et al., 2020) [12].

From 2020 to 2021, amidst the coronavirus-19 (COVID-19) pandemic, malaria emerged as the sixth leading cause of mortality among the hospitalized patients admitted to HCL. Furthermore, in 2021, COVID-19 remained the primary cause for medical consultations, indicating a notable rise in the fatality rate compared to 2020 (increasing from 2% to 4%; HCL internal Official Report). According to an HCL internal Official Report, the total number of cases (per year) of malaria had a notable surge, rising from 1640 in 2021 to 2273 in 2022. This might be due to the constrained availability of healthcare services and the heightened level of fear within the population regarding seeking medical help [13]. Also, the decreased income of families due to COVID-19 pandemic restrictions most probably led to increased challenges in covering health-related costs [14].

The number of severe malaria cases at HCL nearly tripled from 2021 (167 cases) to 2022 (473 cases), mirroring trends seen in other African studies [13]. Despite the increase in case numbers, the mortality rate decreased from 10.2% in 2021 to 5.9% in 2022. This is lower than the mortality rates typically reported in severe malaria cases, which is usually well over 5% [5,15]. Several factors affecting the prognosis of *P. falciparum* malaria have been identified [14]. The SOFA, a score that describes quantitively the degree of organ dysfunction/failure over time in patients, has become a common feature for the assessment of morbidity in critical illness [16] including severe malaria [17]. An increasing SOFA score reflects the severity of illness [12,16,18,19]. Although the median SOFA score was 11 (range of 4–21), reflecting severe illness in ICU patients, it was not a reliable predictor of mortality in our study, which aligns with findings from Luanda, Angola [12]. We could not corroborate that the SOFA is a good predictor of mortality in the ICU [20]. This could be due to the small number of ICU patients who were non-survivors, as 68% of ICU deaths occurred within 48 h of admission, limiting the predictive value of the SOFA score for 28-day mortality [19,20].

Mortality due to severe malaria is influenced by many factors, including host immunity [5]. The *P. falciparum* malaria positivity rate tends to be higher during productive age [17,21], although the severity of the disease tends to be lower, due to higher antimalarial immunity [6]. In our study, the majority of patients were young adults (mean of 29.5 ± 13.6 years), closely mirroring the demographic profile of those who died (28.9 ± 14.1 years). Recent studies suggest that severe and complicated malaria is more common in this age group than previously thought, highlighting the need for further research into the underlying causes of this trend [8,22]. The findings of our study are consistent with previous research, indicating that hyperparasitemia alone is not a reliable predictor of outcomes in severe malaria. This underscores the importance of assessing a combination of clinical and laboratory markers for a comprehensive evaluation of patient risk. While hyperparasitemia (parasites/µL) may not directly predict mortality [5], it does contribute to the risk of severe anemia, which can exacerbate the severity of illness as the disease advances [23].

In our study, renal impairment was the most prevalent clinical feature overall, followed by impaired consciousness and jaundice. These findings align with other studies from sub-Saharan Africa [8,9,22], demonstrating similarities in clinical presentations, with the exception of jaundice, which was more frequent in our cohort. Despite a decrease in overall mortality, severe malaria continues to significantly impact young adults in Huíla, contributing to substantial mortality rates.

The statistical analysis performed in our study revealed that renal impairment, metabolic acidosis, impaired consciousness, multiple convulsions, hypoglycemia, and bleeding were significantly associated with higher mortality, while jaundice, hyperparasitemia, and shock were not. Renal impairment was observed in 76.7% of non-survivors compared to 37.4% of survivors, emphasizing its significant association with poor outcomes. Similarly, metabolic acidosis was present in 52.6% of non-survivors compared to only 7.5% of survivors, indicating that this criterion is a strong marker for mortality risk. Impaired consciousness affected 42.2% of non-survivors compared to 10.6% of survivors, underscoring its association with higher fatality rates. In addition, multiple convulsions, hypoglycemia, and bleeding were strongly associated with mortality. Patients experiencing multiple convulsions had a more than threefold increased risk of death, highlighting the importance of monitoring and managing this condition. Hypoglycemia showed an even stronger association, affecting 12.2% of non-survivors versus 1.4% of survivors, indicating that patients presenting with hypoglycemia were 15 times more likely to succumb to severe malaria. Bleeding emerged as one of the most significant indicators of poor prognosis, detected in 24.4% of non-survivors and only 1.8% of survivors, highlighting the need for immediate management to improve patient outcomes. Despite being observed in a substantial proportion of non-survivors, jaundice did not show a significant difference when compared to survivors. Hyperparasitemia was present at similar rates in both groups and did not emerge as a significant predictor of mortality. Shock also showed no meaningful association with mortality, suggesting that its presence did not significantly impact survival outcomes in this cohort. These findings emphasize the urgent need for targeted strategies that focus on the early detection and management of key mortality predictors, such as renal impairment, metabolic acidosis, impaired consciousness, hypoglycemia, and bleeding. Enhanced prevention, surveillance, and case management in Huíla Province are crucial for reducing the morbidity and mortality associated with severe malaria. This study’s results align with other research from sub-Saharan Africa, confirming these severe malaria manifestations as pivotal indicators of patient outcomes [24].

Among patients with renal impairment, jaundice was the second most common complication, affecting 27.3% in 2021 and 47.6% in 2022. While jaundice has been associated with renal impartment in other studies [8], the higher incidence at HCL is nevertheless worrisome. Impaired consciousness, another common symptom in our study, is often linked to cerebral malaria. The long-term effects attributed to cerebral malaria (impaired consciousness) are extensively studied in pediatric patients [25,26] and thought to be more frequent in children than in adults [27,28]. In fact, in African children, severe malaria is the leading cause of acquired neurodisability [29]. However, studies on neurological sequelae in adults, particularly those involving serial follow-up assessments, are currently lacking. Our study was not designed to identify the cause of the increased number of patients presenting renal impairment and impaired consciousness from 2021 to 2022, but it highlighted the need to understand the reason behind this increase in order to enable mitigation measures to be implemented at HCL, more so because an emerging link between acute renal impairment and the brain (neurologic deficits and neurocognitive sequelae) in severe malaria patients is gaining momentum [30,31,32,33,34,35]. Rapid diagnosis with timely blood transfusion, renal replacement therapy, and restrictive fluid therapy can improve survival in severe malaria [36].

Thrombocytopenia is a common finding in adults with severe *P. falciparum* malaria and is often seen as a potential predictor of poor outcomes [37,38]. In both survivors and the non-survivor group in our study, we observed a tendency for the platelet count to decrease as parasitemia increased (Supplementary Figure S2), in line with other findings [37,38,39,40]. Hence, we agree that thrombocytopenia might be an indicator of disease severity in adults with *P. falciparum* malaria, but has limited utility in prognostication, triage, and management [39,40].

The time between symptom onset to hospital admission, self-reported by patients, showed no difference between 2021 (4.6 ± 2.2 days) and 2022 (4.5 ± 2.1 days). This delay in seeking care may not fully explain the increased severity of illness in 2022. However, significant changes in patient management and healthcare restructuring at HCL likely improved patient outcomes. Well-equipped ICUs with trained staff tend to produce better outcomes compared to peripheral healthcare facilities [41,42]. The increase in patient admissions in 2022 also provided a broader statistical base, which may have contributed to the reduction in mortality by stabilizing the dataset and mitigating irregularities that can affect smaller groups. Thus, the combination of increased admissions and enhanced ICU conditions due to recent infrastructural improvements by the hospital (HCL) likely played a pivotal role in the substantial reduction in mortality rates observed in 2022.

This retrospective study is subject to limitations. As a single-center retrospective study, the generalizability of our findings to other settings may be limited. Another limitation is the lack of systematically recorded data on antimalarial treatment administered before and during transportation, as well as the time-lapse from symptom onset to hospital admission (it was not in the study design). These could impact negatively on the severity of symptoms observed upon hospital presentation. Nevertheless, this study provides both a baseline and valuable (up-to-date) data on severe malaria in adult patients in a low-transmission region. A well-designed cohort study with serial follow-up assessments in adults is needed to further investigate the clinical outcomes and long-term effects of severe malaria.

## 5. Conclusions

Although the total number of severe malaria patients in 2022 almost tripled in comparison to 2021 (473 and 167, respectively), the mortality rate was reduced by half. The increase in malaria cases during 2022 underscores the need for the enhanced monitoring and surveillance of malaria in subsequent years. It is crucial to assess the long-term impact of reduced malaria prevention measures during the COVID-19 pandemic, as well as the potential emergence of antimalarial drug resistance. The increase in the prevalence of renal impairment observed from 2021 to 2022 highlights a key area for further investigation. This study provides a strong foundation for future research in low-transmission regions and offers a valuable reference for ongoing malaria studies in Angola.

## Figures and Tables

**Table 1:** Demographics of the patients admitted to HCL in 2021 and 2022 during the peak months of malaria transmission. MED, Internal Medicine; INF, Infectiology; ICU, Intensive Care Unit.

		2021				2022				Total %
		ICU %	MED %	INF %	Total %	ICU %	MED %	INF %	Total %	
**Sex**	Female	19.2 (5/26)	47.6 (59/124)	52.9 (9/17)	43.7 (73/167)	21.8 (12/55)	44.9 (150/334)	29.8 (25/84)	39.5 (187/473)	40.6 (260/640)
	Male	80.8 (21/26)	52.4 (65/124)	47.1 (8/17)	56.3 (94/167)	78.2 (43/55)	55.1 (184/334)	70.2 (59/84)	60.5 (286/473)	59.4 (380/640)
**Age group**	14–18	7.7 (2/26)	15.3 (19/124)	5.9 (1/17)	13.2 (22/167)	12.7 (7/55)	11.4 (38/334)	19.0 (16/84)	12.9 (61/473)	13.0 (83/640)
	18–60	84.6 (22/26)	78.2 (97/124)	88.2 (15/17)	80.2 (134/167)	83.7 (46/55)	82.0 (274/334)	75.0 (63/84)	81.0 (383/473)	80.8 (517/640)
	≥60	7.7 (2/26)	6.5 (8/124)	5.9 (1/17)	6.6 (11/167)	3.6 (2/55)	6.6 (22/334)	6.0 (5/84)	6.1 (29/473)	6.2 (40/640)

**Table 2:** Severe malaria criteria comparison between all patients, survivors and non-survivors, across all departments (the ICU, MED, and INF) during the study period at the Hospital Central Dr. António Agostinho Neto of Lubango. The *p*-values were calculated to assess the statistical significance of differences between survivors and non-survivors. Impaired consciousness: Glasgow coma score (GCS) < 11; multiple convulsions: more than two episodes within 24 h; metabolic acidosis: base deficit > 8 mEq/L, or if unavailable, plasma bicarbonate < 15 mmol/L or venous plasma lactate ≥ 5 mmol/L; hypoglycemia: blood glucose < 40 mg/dL; anemia: hemoglobin < 7 g/dL and/or hematocrit < 20%, with a parasite count > 10,000/μL; renal impairment: plasma or serum creatinine > 3 mg/dL and/or blood urea > 20 mmol/L; jaundice: bilirubin > 3 mg/dL; hyperparasitemia: parasite density > 500,000 parasites/μL; shock: systolic blood pressure < 80 mmHg. Bleeding: recurrent or prolonged bleeding from the nose, gums, or venipuncture sites; hematemesis or melaena.

Severe Malaria Criteria		All Patients (*n* = 640)	Survivors (*n* = 595)	Non-Survivors (*n* = 45)	*p*-Value
**Age (Mean ± SD)**		29.5 ± 13.6	30.3 ± 14.7	28.9 ± 14.1	0.33
**Sex**	**Female**	260/640 40.6%	240/595 40.3%	20/45 44.4%	
	**Male**	380/640 59.4%	355/595 59.7%	25/45 55.6%	
**Impaired consciousness**		68/640 10.6%	49/595 8.25%	19/45 42.2%	<0.001
**Multiple convulsions**		32/640 5.0%	26/595 4.4%	6/45 13.3%	0.019
**Metabolic acidosis**		38/640 5.9%	18/595 3.0%	20/41 48.8%	<0.001
**Hypoglycemia**		11/471 2.3%	6/430 1.4%	5/41 12.2%	<0.001
**Anemia**	**Hemoglobin**	74/639 11.6%	66/594 11.1%	8/45 17.8%	
	**Hematocrit**	78/638 12.2%	69/593 11.6%	9/45 20.0%	
	**Overall**	84/640 13.1%	74/595 12.4%	10/45 22.2%	0.068
**Renal impairment**	**Creatinine**	84/547 15.4%	72/509 14.1%	12/38 31.6%	
	**Blood urea**	205/559 36.7%	172/519 33.1%	33/40 82.5%	
	**Overall**	209/559 37.4%	176/516 34.1%	33/43 76.7%	<0.001
**Jaundice**		157/544 28.9%	143/509 28.1%	14/35 40.0%	0.175
**Hyperparasitemia**		40/571 7.0%	38/529 1.3%	2/42 4.8%	0.78
**Shock**		62/637 9.7%	59/592 9.9%	3/45 6.7%	0.61
**Bleeding**		22/640 3.4%	11/595 1.8%	11/45 24.4%	<0.001

## Data Availability

This study adheres to strict ethical standards, ensuring compliance with recognized guidelines for scientific competence and ethical conduct. All data collected have been anonymized and coded to guarantee participant privacy and confidentiality. Due to ethical and privacy restrictions, the raw data used in this study is not publicly available. However, aggregated data supporting the reported results can be made available upon reasonable request to the corresponding author, in accordance with the ethical framework of this research.

## Supplementary Materials

The following supporting information can be downloaded at https://www.mdpi.com/article/10.3390/biomedicines12112639/s1: Figure S1—Number of patients admitted to the Central Hospital of Lubango’s Intensive Care Unit (ICU), Internal Medicine, and Infectiology departments during the peak months (January, February, March) of malaria transmission in this region in 2021 and 2022. Supplementary Figure S2—Platelet count (platelets/µL blood) versus parasitemia (parasites/µL blood). (A) Survivors group; (B) non-survivors group. Table S1—Severe malaria profile of patients (regarding severe malaria manifestations) admitted to HCL during 2021 and 2022. The first line displays the number of patients for each department and year. If data were missing for some patients, the percentage of patients meeting the specific severe malaria criteria was calculated based on the available patient count mentioned in the corresponding table cell. Table S2—Profile of patients (regarding severe malaria manifestations) who died during the study period at the Hospital Central Dr. António Agostinho Neto of Lubango. The first line displays the number of patients for each department and year. If data were missing for some patients, the percentage of patients meeting the specific severe malaria criteria was calculated based on the available patient count mentioned in the corresponding table cell.

## Appendix A

A retrospective study was performed in adult patients with severe malaria hospitalized in the departments of Internal Medicine (MED), Infectiology (INF), and the Intensive Care Unit (ICU). This study was based on individual patient records of those admitted to HCL from January to March in 2021 and 2022. The following criteria were used for inclusion in the study: patients of all genders, being at least 14 years of age and without an upper age limit, and a laboratory-confirmed diagnosis of *P. falciparum* infection through a rapid diagnostic test (RDT) or a positive blood smear. For epidemiological purposes, severe malaria was defined according to the WHO, following criteria for severe malaria [1].

For epidemiological purposes, severe malaria was defined according to the World Health Organization [1], indicating the presence of *P. falciparum* infection and one or more of the following criteria:

- Impaired consciousness (Glasgow score, GCS < 11); prostration (generalized weakness so that the person is unable to sit, stand, or walk without assistance);
- Multiple convulsions (more than two episodes within 24 h);
- Hypoglycemia (blood or plasma glucose < 40 mg/dL);
- Anemia (hemoglobin concentration < 7 g/dL or a hematocrit of <20% in adults, with a parasite count > 10,000/µL of blood);
- Renal impairment (plasma or serum creatinine > 3 mg/dL or blood urea > 20 mmol/L);
- Jaundice (plasma or serum bilirubin > 3 mg/dL, with a parasite count > 10,000/µL);
- Pulmonary edema (radiologically confirmed or oxygen saturation < 92% in room air with a respiratory rate > 30/min, often with chest indrawing and crepitations on auscultation);
- Significant bleeding (recurrent or prolonged bleeding from the nose, gums, or venipuncture sites; hematemesis or melaena);
- Shock (compensated shock: capillary refill ≥ 3 s or temperature gradient on leg (mid to proximal limb); no hypotension);
- Decompensated shock (systolic blood pressure < 80 mmHg in adults, with evidence of impaired perfusion);
- Hyperparasitemia (10% or >500,000 parasites/μL);
- Metabolic acidosis (a base deficit of >8 mEq/L or, if not available, a plasma bicarbonate level of <15 mmol/L or venous plasma lactate ≥ 5 mmol/L).

## References

[1] World Health Organization (2023). World Malaria Report 2023. https://www.who.int/publications/i/item/9789240086173.

[2] Huntley B.J., Huntley B.J., Russo V., Lages F., Ferrand N. (2019). Angola in Outline: Physiography, Climate and Patterns of Biodiversity. Biodiversity of Angola: Science & Conservation: A Modern Synthesis.

[3] Tavares W., Morais J., Martins J.F., Scalsky R.J., Stabler T.C., Medeiros M.M., Fortes F.J., Arez A.P., Silva J.C. (2022). Malaria in Angola: Recent progress, challenges and future opportunities using parasite demography studies. Malar. J..

[4] World Health Organization (2023). WHO Guidelines for Malaria, 16 October 2023. World Health Organization. https://iris.who.int/bitstream/handle/10665/373339/WHO-UCN-GMP-2023.01-Rev.1-eng.pdf?sequence=1.

[5] White N.J. (2022). Severe malaria. Malar. J..

[6] Griffin J.T., Hollingsworth T.D., Reyburn H., Drakeley C.J., Riley E.M., Ghani A.C. (2015). Gradual acquisition of immunity to severe malaria with increasing exposure. Proc. R. Soc. B Biol. Sci..

[7] Reyburn H., Mbatia R., Drakeley C., Bruce J., Carneiro I., Olomi R., Cox J., Nkya W.M., Lemnge M., Greenwood B.M. (2005). Association of transmission intensity and age with clinical manifestations and case fatality of severe *Plasmodium falciparum* malaria. JAMA.

[8] Bittaye S.O., Jagne A., Jaiteh L.E., Nadjm B., Amambua-Ngwa A., Sesay A.K., Singhateh Y., Effa E., Nyan O., Njie R. (2022). Clinical manifestations and outcomes of severe malaria in adult patients admitted to a tertiary hospital in the Gambia. Malar. J..

[9] Yusuph R., Sawe H.R., Nkondora P.N., Mfinanga J.A. (2019). Profile and outcomes of patients with acute complications of malaria presenting to an urban emergency department of a tertiary hospital in Tanzania. BMC Res. Notes.

[10] Francisco N.M., van Wyk S., Moir M., San J.E., Sebastião C.S., Tegally H., Xavier J., Maharaj A., Neto Z., Afonso P. (2023). Insights into SARS-CoV-2 in Angola during the COVID-19 peak: Molecular epidemiology and genome surveillance. Influenza Other Respir. Viruses.

[11] Gyeltshen D., Musa S.S., Amesho J.N., Ewelike S.C., Bayoh A.V.S., Al-Sammour C., Camua A.A., Lin X., Lowe M., Ahmadi A. (2021). COVID-19: A novel burden on the fragile health system of Angola. J. Glob. Health.

[12] Antunes M.L., Seixas J., Ferreira H.E., Silva M.S. (2020). Adequacy of Severe Malaria Markers and Prognostic Scores in an Intensive Care Unit in Luanda, Angola: A Clinical Study. J. Clin. Med..

[13] United Nations Conference on Trade and Development (2022). Economic and Social Impact of COVID-19 in Angola 2021. https://unctad.org/system/files/official-document/aldcinf2021d6_en.pdf.

[14] Awucha N.E., Janefrances O.C., Meshach A.C., Henrietta J.C., Daniel A.I., Chidiebere N.E. (2020). Impact of the COVID-19 Pandemic on Consumers’ Access to Essential Medicines in Nigeria. Am. J. Trop. Med. Hyg..

[15] Bittaye S.O., Jagne A., Jaiteh L.E.S., Amambua-Ngwa A., Sesay A.K., Ekeh B., Nadjm B., Ramirez W.E., Ramos A., Okeahialam B. (2023). Malaria in adults after the start of COVID-19 pandemic: An analysis of admission trends, demographics, and outcomes in a tertiary hospital in the Gambia. Malar. J..

[16] Srinamon K., Watson J.A., Silamut K., Intharabut B., Phu N.H., Diep P.T., Lyke K.E., Fanello C., von Seidlein L., Chotivanich K. (2022). The prognostic and diagnostic value of intraleukocytic malaria pigment in patients with severe falciparum malaria. Nat. Commun..

[17] Debash H., Bisetegn H., Ebrahim H., Tilahun M., Dejazmach Z., Getu N., Feleke D.G. (2023). Burden and seasonal distribution of malaria in Ziquala district, Northeast Ethiopia: A 5-year multi-centre retrospective study. BMJ Open.

[18] Lie K.C., Lau C.-Y., Chau N.V.V., West T.E., Limmathurotsakul D., for Southeast Asia Infectious Disease Clinical Research Network (2018). Utility of SOFA score, management and outcomes of sepsis in Southeast Asia: A multinational multicenter prospective observational study. J. Intensiv. Care.

[19] Teparrukkul P., Hantrakun V., Imwong M., Teerawattanasook N., Wongsuvan G., Day N.P., Dondorp A.M., West T.E., Limmathurotsakul D. (2019). Utility of qSOFA and modified SOFA in severe malaria presenting as sepsis. PLoS ONE.

[20] Rudd K.E., Seymour C.W., Aluisio A.R., Augustin M.E., Bagenda D.S., Beane A., Byiringiro J.C., Chang C.-C.H., Colas L.N., Day N.P.J. (2018). Association of the Quick Sequential (Sepsis-Related) Organ Failure Assessment (qSOFA) Score with Excess Hospital Mortality in Adults with Suspected Infection in Low- and Middle-Income Countries. JAMA.

[21] Nkumama I.N., O’meara W.P., Osier F.H. (2016). Changes in Malaria Epidemiology in Africa and New Challenges for Elimination. Trends Parasitol..

[22] Boushab B.M., Salem M.S.O.A., Boukhary A.O.M.S., Parola P., Basco L. (2020). Clinical Features and Mortality Associated with Severe Malaria in Adults in Southern Mauritania. Trop. Med. Infect. Dis..

[23] Poespoprodjo J.R., Douglas N.M., Ansong D., Kho S., Anstey N.M. (2023). Malaria. Lancet.

[24] Plucinski M.M., Ferreira M., Ferreira C.M., Burns J., Gaparayi P., João L., da Costa O., Gill P., Samutondo C., Quivinja J. (2017). Evaluating malaria case management at public health facilities in two provinces in Angola. Malar. J..

[25] Storm J., Jespersen J.S., Seydel K.B., Szestak T., Mbewe M., Chisala N.V., Phula P., Wang C.W., Taylor T.E., Moxon C.A. (2019). Cerebral malaria is associated with differential cytoadherence to brain endothelial cells. EMBO Mol. Med..

[26] Kariuki S.M., Abubakar A., Newton C.R., Kihara M. (2014). Impairment of executive function in Kenyan children exposed to severe falciparum malaria with neurological involvement. Malar. J..

[27] Boivin M.J., Bangirana P., Byarugaba J., Opoka R.O., Idro R., Jurek A.M., John C.C. (2007). Cognitive impairment after cerebral malaria in children: A prospective study. Pediatrics.

[28] Birbeck G.L., Beare N., Lewallen S., Glover S.J., Molyneux M.E., Kaplan P.W., Taylor T.E. (2010). Identification of malaria retinopathy improves the specificity of the clinical diagnosis of cerebral malaria: Findings from a prospective cohort study. Am. J. Trop. Med. Hyg..

[29] Idro R., Marsh K., John C.C., Newton C.R.J. (2010). Cerebral Malaria: Mechanisms of Brain Injury and Strategies for Improved Neurocognitive Outcome. Pediatr. Res..

[30] Conroy A.L., Opoka R.O., Bangirana P., Idro R., Ssenkusu J.M., Datta D., Hodges J.S., Morgan C., John C.C. (2019). Acute kidney injury is associated with impaired cognition and chronic kidney disease in a prospective cohort of children with severe malaria. BMC Med..

[31] Namazzi R., Opoka R., Datta D., Bangirana P., Batte A., Berrens Z., Goings M.J., Schwaderer A.L., Conroy A.L., John C.C. (2022). Acute Kidney Injury Interacts with Coma, Acidosis, and Impaired Perfusion to Significantly Increase Risk of Death in Children with Severe Malaria. Clin. Infect. Dis..

[32] Namazzi R., Batte A., Opoka R.O., Bangirana P., Schwaderer A.L., Berrens Z., Datta D., Goings M., Ssenkusu J.M., Goldstein S.L. (2022). Acute kidney injury, persistent kidney disease, and post-discharge morbidity and mortality in severe malaria in children: A prospective cohort study. eClinicalMedicine.

[33] Conroy A.L., Datta D., Hoffmann A., Wassmer S.C. (2023). The kidney–brain pathogenic axis in severe falciparum malaria. Trends Parasitol..

[34] Hickson M.R., Conroy A.L., Bangirana P., Opoka R.O., Idro R., Ssenkusu J.M., John C.C. (2019). Acute kidney injury in Ugandan children with severe malaria is associated with long-term behavioral problems. PLoS ONE.

[35] Bangirana P., Conroy A.L., Opoka R.O., Hawkes M.T., Hermann L., Miller C., Namasopo S., Liles W.C., John C.C., Kain K.C. (2018). Inhaled nitric oxide and cognition in pediatric severe malaria: A randomized double-blind placebo controlled trial. PLoS ONE.

[36] Xu J.-W., Deng D.-W., Wei C., Zhou X.-W., Li J.-X. (2023). Treatment-seeking behaviours of malaria patients versus non-malaria febrile patients along China-Myanmar border. Malar. J..

[37] Sirak S., Fola A.A., Worku L., Biadgo B. (2016). Malaria parasitemia and its association with lipid and hematological parameters among malaria-infected patients attending at Metema Hospital, Northwest Ethiopia. Pathol. Lab. Med. Int..

[38] Lampah D.A., Yeo T.W., Malloy M., Kenangalem E., Douglas N.M., Ronaldo D., Sugiarto P., Simpson J.A., Poespoprodjo J.R., Anstey N.M. (2014). Severe malarial thrombocytopenia: A risk factor for mortality in Papua, Indonesia. J. Infect. Dis..

[39] Hanson J., Phu N.H., Hasan M.U., Charunwatthana P., Plewes K., Maude R.J., Prapansilp P., Kingston H.W., Mishra S.K., Mohanty S. (2015). The clinical implications of thrombocytopenia in adults with severe falciparum malaria: A retrospective analysis. BMC Med..

[40] Tiiba J.-D.I., Ahmadu P.U., Naamawu A., Fuseini M., Raymond A., Osei-Amoah E., Bobrtaa P.C., Bacheyie P.P., Abdulai M.A., Alidu I. (2022). Thrombocytopenia a predictor of malaria: How far?. J. Parasit. Dis..

[41] Legros F., Bouchaud O., Ancelle T., Arnaud A., Cojean S., Le Bras J., Danis M., Fontanet A., Durand R. (2007). Risk factors for imported fatal *Plasmodium falciparum* malaria, France, 1996–2003. Emerg. Infect. Dis..

[42] Njuguna P., Newton C. (2004). Management of severe falciparum malaria. J. Postgrad. Med..

