# Supplementary material for: Severe Malaria in Angola: The Clinical Profile and Disease Outcome Among Adults from a Low-Endemic Area
Source: Biomedicines. 2024 Nov 19;12(11):2639. doi: 10.3390/biomedicines12112639 (PMC11592004; doi:10.3390/biomedicines12112639)
Supplement: Supplementary file 1 [file biomedicines-12-02639-s001.zip › Supplementary material_Figure S1_Morais 2024.pdf]

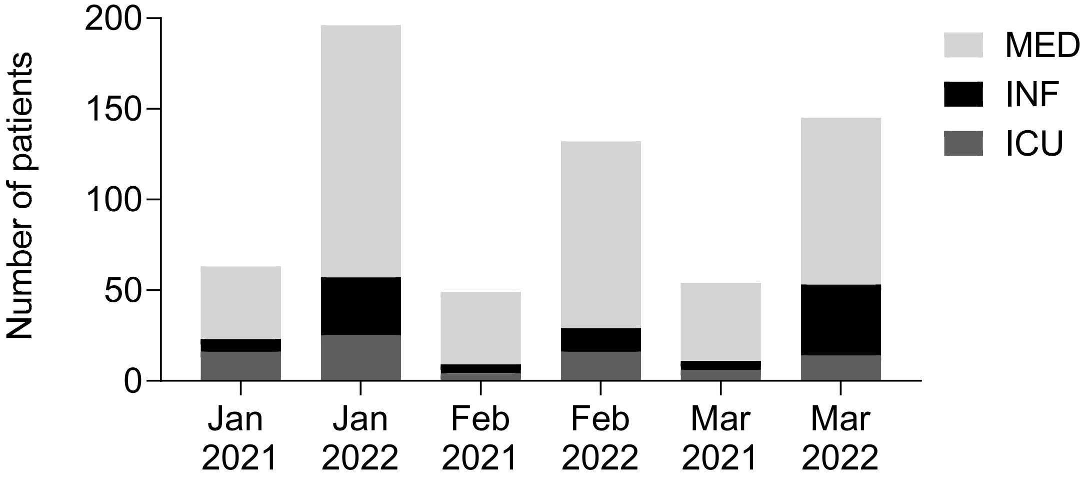

Supplementary Figure S1 – Number of patients admitted to the Central Hospital of Lubango's Intensive Care Unit (ICU), Internal Medicine, and Infectiology departments during the peak months (January, February, March) of malaria transmission in this region in 2021 and 2022.
