# Supplementary material for: Severe Malaria in Angola: The Clinical Profile and Disease Outcome Among Adults from a Low-Endemic Area
Source: Biomedicines. 2024 Nov 19;12(11):2639. doi: 10.3390/biomedicines12112639 (PMC11592004; doi:10.3390/biomedicines12112639)
Supplement: Supplementary file 1 [file biomedicines-12-02639-s001.zip › Supplementary material_Figure S2_Morais 2024.pdf]

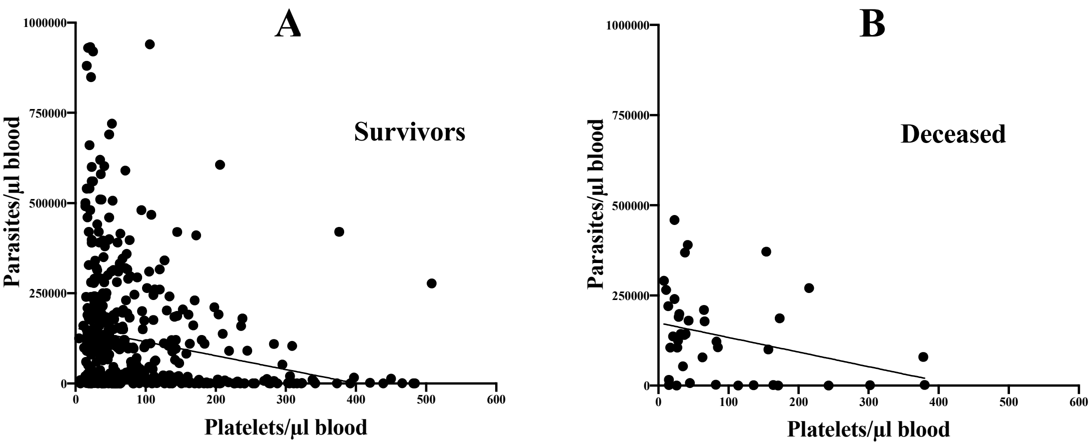

Supplementary Figure S2 – Platelets count (platelets/ µl blood) versus parasitemia (parasites/µl blood). A survivors group; **B** non-survivors group.
