# Supplementary material for: Severe Malaria in Angola: The Clinical Profile and Disease Outcome Among Adults from a Low-Endemic Area
Source: Biomedicines. 2024 Nov 19;12(11):2639. doi: 10.3390/biomedicines12112639 (PMC11592004; doi:10.3390/biomedicines12112639)
Supplement: Supplementary file 1 [file biomedicines-12-02639-s001.zip › Supplementary material_Table S1_Morais 2024.pdf]

Supplementary Table S1 – Severe malaria profile of patients (regarding severe malaria manifestations) admitted to HCL during 2021 and 2022. The first row enumerates the patient counts per department and year. Where data were missing, percentages representing the proportion of patients exhibiting specific severe malaria manifestations were calculated based on the patient count available in the corresponding table cell. Impaired Consciousness: Glasgow Coma Score (GCS) < 11; Multiple Convulsions: More than two episodes within 24 hours; Metabolic Acidosis: Base deficit > 8 mEq/L, or if unavailable, plasma bicarbonate < 15 mmol/L or venous plasma lactate ≥ 5 mmol/L; Hypoglycemia: Blood glucose < 40 mg/dL; Anemia: Hemoglobin < 7 g/dL and/or Hematocrit < 20%, with a parasite count > 10,000/µL; Renal Impairment: Plasma or serum creatinine > 3 mg/dL and/or blood urea > 20 mmol/L; Jaundice: Bilirubin > 3 mg/dL; Hyperparasitemia: Parasite density > 500,000 parasites/µL; Shock: Systolic blood pressure < 80 mmHg; Bleeding: Recurrent or prolonged bleeding from the nose, gums, or venipuncture sites; hematemesis or melaena; The total number of patients (n) in the denominator varies across different criteria, as some patients did not have records for certain criteria. As a result, they were excluded from the total count and percentage calculation for that specific criterion.

		2021			2022			
**Severe malaria criteria**		**ICU**	**MED**	**INF**	**ICU**	**MED**	**INF**	**Total (n=640)**
**Impaired Consciousness**		16/26<br>61.5%	8/124<br>6.5%	0/17<br>0.0%	30/55<br>54.5%	10/334<br>3.0%	4/84<br>4.8%	68/640<br>10.6%
**Multiple Convulsions**		6/26<br>23.1%	5/124<br>4.0%	0/17<br>0.0%	6/55<br>10.9%	13/334<br>3.9%	2/84<br>2.4%	32/640<br>5.0%
**Metabolic Acidosis**		9/26<br>34.6%	2/124<br>1.6%	0/17<br>0.0%	27/55<br>49.1%	0/334<br>0.0%	0/84<br>0.0%	38/640<br>5.9%
**Hypoglycemia**		2/22<br>9.1%	1/79<br>1.3%	0/9<br>0.0%	3/50<br>6.0%	5/262<br>1.9%	0/49<br>0.0%	11/471<br>2.3%
**Anemia**	Hemoglobin	4/26<br>15.4%	13/124<br>10.5%	1/17<br>5.9%	9/55<br>16.4%	34/334<br>10.2%	13/83<br>15.7%	74/639<br>11.6%
	Hematocrit	5/26<br>19.2%	15/123<br>12.2%	1/17<br>5.9%	9/55<br>16.4%	35/334<br>10.5%	13/83<br>15.7%	78/638<br>12.2%
	Overall	5/26<br>19.2%	15/124<br>12.1%	1/17<br>5.9%	11/55<br>20.0%	38/334<br>11.4%	14/84<br>16.7%	84/640<br>13.1%
**Renal Impairment**	Creatinine	8/24<br>33.3%	4/98<br>4.1%	0/11<br>0.0%	18/49<br>36.7%	43/292<br>14.7%	11/73<br>15.1%	84/547<br>15.4%
	Blood Urea	14/25<br>56.0%	19/98<br>19.4%	4/11<br>36.4%	43/55<br>78.2%	99/295<br>33.6%	26/75<br>34.7%	205/559<br>36.7%
	Overall	14/25<br>56.0%	20/98<br>20.4%	4/11<br>36.4%	43/55<br>78.2%	101/295<br>34.2%	27/75<br>36.0%	209/559<br>37.4%
**Jaundice**		11/23<br>47.8%	20/95<br>21.1%	3/10<br>30.0%	28/51<br>54.9%	74/293<br>25.3%	21/72<br>29.2%	157/544<br>28.9%
**Hyperparasitemia**		2/24<br>8.3%	7/104<br>6.7%	3/16<br>18.8%	3/51<br>5.9%	23/303<br>7.6%	2/73<br>2.7%	40/571<br>7.0%
**Shock**		3/26<br>11.5%	6/123<br>4.9%	0/16<br>0.0%	3/55<br>5.5%	38/333<br>11.4%	12/84<br>14.3%	62/637<br>9.7%
**Bleeding**		2/26	5/124	0/17	5/55	8/334	2/84	22/640

	7.7%	4.0%	0.0%	9.1%	2.4%	2.4%	3.4%
